# Development of real-time recombinase polymerase amplification assay for rapid and sensitive detection of canine parvovirus 2

**DOI:** 10.1186/s12917-017-1232-z

**Published:** 2017-11-06

**Authors:** Yunyun Geng, Jianchang Wang, Libing Liu, Yan Lu, Ke Tan, Yan-Zhong Chang

**Affiliations:** 1 College of Life Sciences, Hebei Normal University, No.20, Road E. 2nd Ring South, Yuhua District, Shijiazhuang, Hebei Province 050024 People’s Republic of China; 2 Center of Inspection and Quarantine, Hebei Entry-Exit Inspection and Quarantine Bureau, No.318 Hepingxilu Road, Shijiazhuang, Hebei Province 050051 People’s Republic of China; 3 Hebei Academy of inspection and quarantine science and technology, No.318 Hepingxilu Road, Shijiazhuang, Hebei Province 050051 People’s Republic of China

**Keywords:** Canine parvovirus, Exo probe, Recombinase polymerase amplification

## Abstract

**Background:**

Canine parvovirus 2, a linear single-stranded DNA virus belonging to the genus *Parvovirus* within the family *Parvoviridae*, is a highly contagious pathogen of domestic dogs and several wild canidae species. Early detection of canine parvovirus (CPV-2) is crucial to initiating appropriate outbreak control strategies. Recombinase polymerase amplification (RPA), a novel isothermal gene amplification technique, has been developed for the molecular detection of diverse pathogens. In this study, a real-time RPA assay was developed for the detection of CPV-2 using primers and an exo probe targeting the CPV-2 nucleocapsid protein gene.

**Results:**

The real-time RPA assay was performed successfully at 38 °C, and the results were obtained within 4–12 min for 10^5^–10^1^ molecules of template DNA. The assay only detected CPV-2, and did not show cross-detection of other viral pathogens, demonstrating a high level of specificity. The analytical sensitivity of the real-time RPA was 10^1^ copies/reaction of a standard DNA template, which was 10 times more sensitive than the common RPA method. The clinical sensitivity of the real-time RPA assay matched 100% (n = 91) to the real-time PCR results.

**Conclusion:**

The real-time RPA assay is a simple, rapid, reliable and affordable method that can potentially be applied for the detection of CPV-2 in the research laboratory and point-of-care diagnosis.

**Electronic supplementary material:**

The online version of this article (10.1186/s12917-017-1232-z) contains supplementary material, which is available to authorized users.

## Background

Canine parvovirus diseases, caused by canine parvovirus type 2 (CPV-2), is highly contagious and prevalent worldwide in domestic and wild canids. CPV-2 emerged as a novel pathogen in 1978 and spread rapidly worldwide [1, 2]. Within a few years, the original type 2 virus underwent a rapid and extensive evolution, and was replaced by two antigenic types, termed CPV-2a and CPV-2b. Recently, a new antigenic variant, CPV-2c, was reported in dogs [3]. CPV-2 is a small non-enveloped, linear single-stranded DNA virus belonging to the family *Parvoviridae*, genus *Parvovirus*, and is considered as a major cause for large numbers of animal deaths worldwide [4]. CPV2-infected dogs are characterized by a gastroenteritis disorder with clinical signs of anorexia, lethargy, vomiting, fever and diarrhea (from mucoid to hemorrhagic) [5, 6]. Because of infection and damage to the bone marrow, parvovirus can also cause acute leukopenia with lymphopenia and neutropenia. When CPV-2 shed into the feces, it can be spread by direct oral or nasal contact. Furthermore, CPV-2 is extremely stable in the environment and can survive for several months. Therefore, early, rapid and accurate diagnosis of CPV-2 infection would help veterinarians to implement appropriate strategies in time to improve disease management and prevent outbreaks, particularly within a shelter environment.

Several traditional diagnosis methods exist for CPV-2, including direct observation by electron microscopy, virus isolation in a suitable cell culture system, serological tests such as the latex agglutination test (LAT), hemaghaemagglutination (HA) test, ELISA and so on. These methods are often time-consuming, laborious, and have low sensitivity [7, 8]. With the advances in molecular detection techniques, a substantial number of gene amplification-based assays have been described for CPV-2 diagnosis such as polymerase chain reaction (PCR), nested PCR, real-time PCR, reverse-transcription loop-mediated isothermal amplification (RT-LAMP) and insulated isothermal PCR (iiPCR) [9–15]. Among these methods, nested PCR, real-time PCR and RT-LAMP have shown high sensitivity. However, due to the requirement of an expensive thermocycler and well-experienced technicians, implementation of these assays is limited in the field and at the point- of -care (POC). The iiPCR method was reported for its sensitive detection of CPV-2, but the reaction time was about 60 min [9]. In addition, the SNAP test based on ELISA protocols for detection of viral antigens is commonly used for CPV-2 diagnosis and can be completed in about 8 min by employing the commercial kit, whereas PCR seems to be more sensitive than SNAP [8, 13, 16, 17]. Recently we reported on recombinase polymerase amplification (RPA) as a rapid, specific, sensitive and cost-effective molecular method for POC diagnosis of CPV-2 infection [18].

RPA is an isothermal gene amplification technique [19]. Similar to conventional PCR, the use of two opposing primers allows exponential amplification of the target sequence in RPA, but the latter is tolerant to 5–9 mismatches in the primer and probe without influencing the performance of the assay. RPA possesses superiority in speed, portability and accessibility compared to PCR, and it has been used to replace the PCR method for the molecular detection of diverse pathogens, such as fungi, parasites, bacteria and viruses [20–22]. Based on our previous study, we developed here a real-time RPA assay for simple, rapid, convenient and POC detection of CPV-2, which utilizes an exo probe and a portable, user-friendly POC tube scanner.

## Methods

### DNA/RNA extraction and RNA reverse transcription

CPV-2a, CPV-2b, CDV, CCoV and CPIV (shown in Table 1) were propagated in Madin–Darby canine kidney (MDCK, CRL-2936™) cells and pseudorabies virus (PRV) in Syrian baby hamster kidney (BHK-21, CCL-10™) cells (ATCC, Manassas, USA). Two hundred microliters of cell culture medium, after brief centrifugation to remove cell debris, was used for viral DNA and RNA extraction using the TIANamp Virus DNA kit (Tiangen Biotech Co., Ltd., Beijing, China) and Trizol reagent (Invitrogen, Beijing, China), respectively. Viral DNA and RNA were quantified using a ND-2000c spectrophotometer (NanoDrop, Wilmington, DE, USA). One hundred nanograms of extracted viral RNA was reverse transcribed to cDNA using the Primescript™ II 1st strand cDNA Synthesis kit (Takara) according to the manufacturer’s instructions. The synthesized cDNA was then purified using the cDNA purification kit (Takara, Dalian, China) and quantified using a ND-2000c spectrophotometer. For viral DNA extraction from the clinical samples, the starting material consisting of 10 mg of feces for each sample was emulsified in 1 mL sterile phosphate-buffered saline (PBS) and centrifuged at 10000 rpm for 10 min at 4 °C. The supernatant was collected and used for viral DNA extraction using the TIANamp Virus DNA kit. Viral DNA extracted from each fecal sample was finally eluted in 20 μl of nuclease-free water. All DNA and cDNA templates were stored at −20 °C until assayed.

**Table 1:** List of Virus strains and clinical samples

Virus/Clinical samples	Strain	Reference
Canine parvovirus type 2a	CPV-b114	
Canine parvovirus type 2b	SJZ101	
Canine distemper virus	CDV-FOX-TA	[8]
Canine coronavirus	VR-809	
Canine parainfluenza virus	CPIV/A-20/8	
Pseudorabies virus	Barth-K61	
91 fecal swab samples^a^		No

^a^Ninety-one fecal swab samples were collected from the dogs sent to our laboratory from 2012 to 2016 and snap-frozen for storage at −80 °C. Seventy-six of the above clinical samples were detected as CPV-2 positive, and fifteen of them were CPV-2 negative by real-time PCR

### Generation of standard DNA

To generate a CPV-2 standard DNA for the real-time RPA, a PCR product containing 1755 bp covering the region of interest of VP2 was amplified from the CPV-2a DNA using VP2-Forward and VP2-Reverse as primers (Table 2) and cloned into the pMD19-T vector using the pMD19-T Vector Cloning Kit (Takara) according to manufacturer’s instructions. The resulting plasmid, pCPV-VP2, was transformed into *Escherichia coli* DH5α cells, and the positive clones were confirmed by sequencing using M13 primers (Invitrogen®, Carlsbad, CA, USA). pCPV-VP2 was purified with the SanPrep Plasmid MiniPrep Kit (Sangon Biotech Co., Ltd., Shanghai, China) and quantified using a ND-2000c spectrophotometer. The copy number of DNA molecules was calculated by the following formula: amount (copies/μL) = [DNA concentration (g/μL) / (plasmid length in base pairs × 660)] × 6.02 × 10^23^.

**Table 2:** Sequences of primers and probes for CPV-2 PCR, real-time PCR and real-time RPA assay

Name	Sequence 5′-3’Amplication size (bp)	
VP2-FP	ATGAGTGATGGAGCAGTTCAACCAGAC	1775
VP2-RP	TTAATATAATTTTCTAGGTGCTAGTTGA	
CPV-FP	AAACAGGAATTAACTATACTAATATATTTA	93
CPV-RP	AAATTTGACCATTTGGATAAACT	
CPV-P	FAM-TGGTCCTTTAACTGCATTAAATAATG TACC-BHQ1	
CPV-RPA-FP	CACTTACTAAGAACAGGTGATGAATTTGCT ACAG	214
CPV-RPA-RP	AGTTTGTATTTCCCATTTGAGTTACACCACGTCT	
CPV-RPA-P	CCTCAAGCTGAAGGAGGTACTAACTTTGGT/ BHQ1-dT//THF //FAM-dT/ATAGGAGTTCAAC AAG-C3 spacer	

### RPA primers and exo probe

The primers used in this study have been described previously [18]. Nucleotide sequences of CPV-2a (GenBank: M24003, AB054215, KF803642), −2b (GenBank: M38245, AY869724, KF803611) and -2c (GenBank: FJ005196, KM236569) were aligned to identify conserved regions of the VP2 gene. Three exo probes were designed based on the conserved region of the VP2 gene. Both primer and probe sequences were 100% identical to their target sequences in the CPV-2a, 2b and 2c genomic DNA. The real-time RPA primers and probes were selected by testing the combination to yield the highest sensitivity (Table 2). Primers and exo probes were synthesized by Sangon Biotech Co., Ltd.

### RPA assay

The real-time RPA reactions were performed in a 50 μL volume using a TwistAmp™ exo kit (TwistDX, Cambridge, UK). Other components included 420 nM each RPA primer, 120 nM exo probe, 14 mM magnesium acetate, and 1 μL of viral or sample DNA. All reagents except for the viral template and magnesium acetate were prepared in a master mix, which was distributed into each 0.2 mL freeze-dried reaction tube containing a dried enzyme pellet. One microliter of viral DNA was added to the tubes. Subsequently, magnesium acetate was pipetted into the tube lids, and then the lids were closed carefully. The magnesium acetate was centrifuged into the rehydrated material using a mini spin centrifuge. After briefly vortexing and centrifuging the reaction tubes once again, they were immediately placed in the Genie III scanner device to start the reaction at 38 °C for 20 min. The fluorescence signal was collected in real-time and increased markedly upon successful amplification.

### Real-time PCR for CPV-2

Real-time PCR specific for CPV-2 was performed on the ABI 7500 instruments described previously with some modifications [18]. Premix Ex Taq™ (Takara Co., Ltd., Dalian, China) was applied in the real-time PCR and the reaction was performed as follows: 95 °C for 3 min, followed by 40 cycles of 95 °C for 10s and 60 °C for 32 s.

### Specificity and analytical sensitivity analysis

Ten nanograms of viral DNA or cDNA was used as the template for the specificity analysis of the real-time RPA assay. The assay was evaluated against a panel of pathogens considered important in dogs, i.e. CPV-2a, CPV-2b, CDV, CCoV, CPIV and PRV.

The recombinant plasmid, pCPV-VP2, was 10-fold serially diluted to achieve DNA concentrations ranging from 10^5^ to 10^0^ copies/μL, which were used as the standard DNA for the CPV-2 RPA sensitivity assay. The real-time RPA was tested using the standard DNA in eight replicates. The threshold time was plotted against the molecules detected.

### Validation with clinical samples

Viral DNA extracted from 91 clinical swab samples were detected by real-time RPA, and the results were compared with those obtained using real-time PCR as previously described [10].

### Statistical methods

For the determination of analytical sensitivity of the real-time PRA assay by the molecular DNA standard, a semi-log regression was calculated using Prism software 5.0 (Graphpad Software Inc., SanDiego, CA). For exact determination, a probit regression was performed using the Statistical Product and Service Solutions software (IBM, Armonk, NY, USA). The evaluation of exo RPA with clinical samples data is presented as R^2^ value.

## Results

**Fig. 1:**
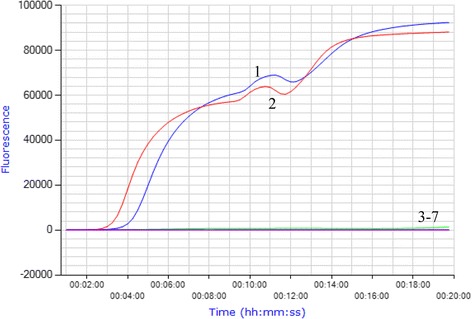
Specificity of the real-time RPA assay for CPV-2 detection. Real-time RPA was carried out at 38 °C for 20 min using 10 ng of viral DNA or cDNA as template. The results showed real-time RPA only amplified the CPV-2a and CPV-2b DNA, but not other viruses tested (*n* = 5). Lane 1, CPV-2a; lane 2, CPV-2b; lane 3, CDV; lane 4, CCoV; lane 5, CPIV; lane 6, PRV; and lane 7, canine genomic DNA

**Fig. 2:**
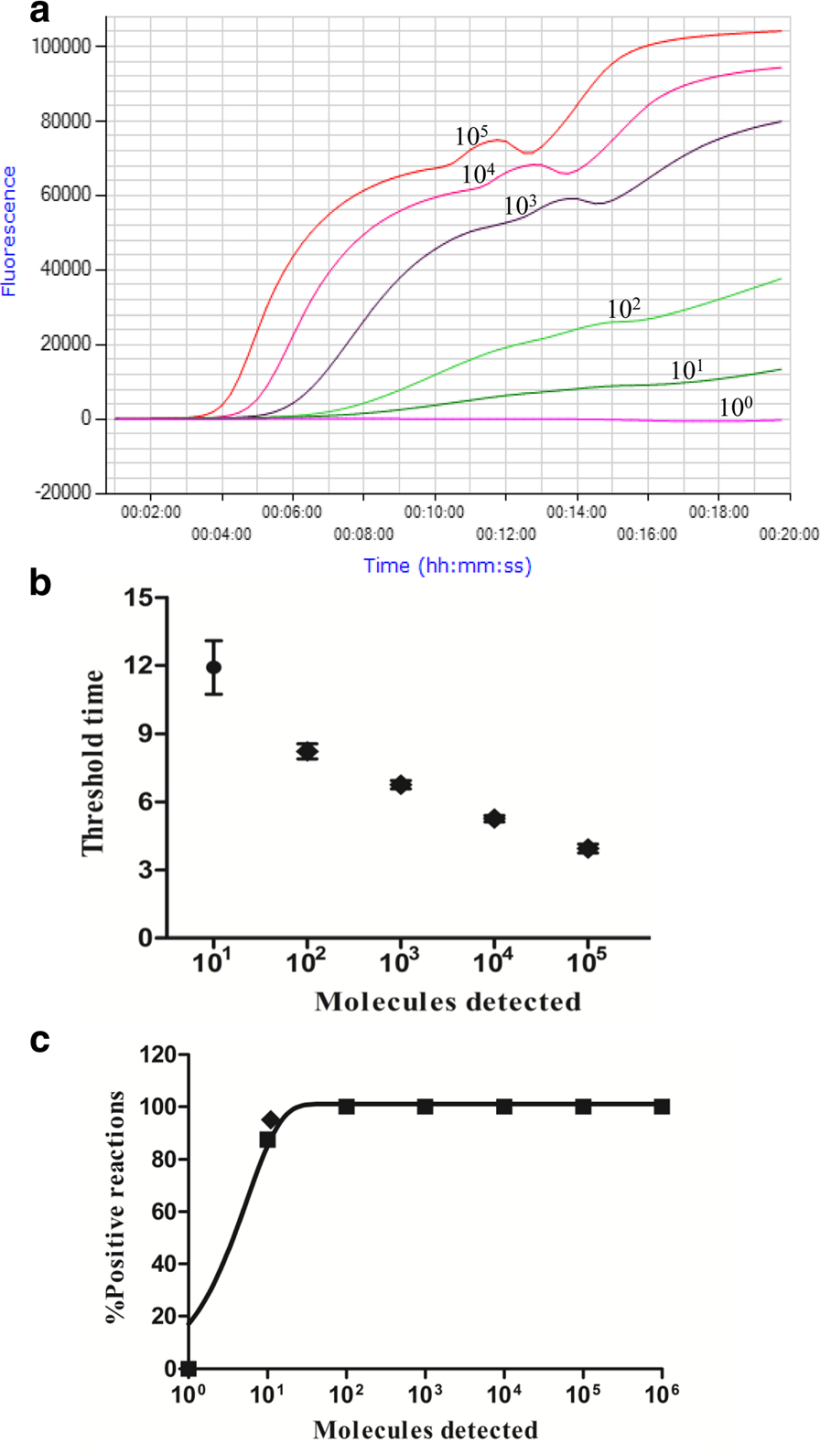
Performance of the CPV-2 real-time RPA. **a** Fluorescence development over time using a dilution range of 10^5^–10^0^ copies of the CPV-2 standard DNA. **b** Semi-logarithmic regression of the data collected from eight CPV-2 real-time RPA test runs on the standard DNA using Prism Software 5.0. Run times of the real-time RPA were 4–12 min to detect 10^5^ and 10^1^ copies, respectively. **c** Probit regression analysis using SPSS software on data from eight runs. The limit of detection of 95% probability (11 copies) is indicated by a rhomboid

The specificity and analytical sensitivity of the real-time RPA assay were analyzed (Figs.1 and 2). Using 10 ng of viral DNA, cDNA or canine genome as template, the results showed that only CPV-2a and CPV-2b were detected by the real-time RPA assay, while the other four viruses, including CDV, CCoV, CPIV and PRV, and canine genome, were not (Fig. 1, *n* = 5). Thus, the real-time RPA assay results demonstrated good specificity for the detection of CPV-2.

To evaluate the sensitivity of the real-time RPA method, a dilution range of 10^5^–10^0^ copies/μL of standard DNA was used as templates, and the real-time RPA and real-time PCR were performed simultaneously. The limit of detection of the real-time RPA was 10^1^ copies (Fig. 2a and b). A probit regression analysis using the results of eight complete molecular standard runs calculated that the limit of detection (LOD) of the real-time RPA was 10^1^ copies per reaction in 95% of cases (Fig. 2c), which was the same as that of the real-time PCR applied in the study (data not shown). With the data from eight runs on the quantitative DNA standards, a semi-log regression analysis for the real-time RPA and real-time PCR was made. Run times of the real-time RPA assay were about 4–12 min for 10^5^–10^1^ copies, respectively (Fig. 2b), while the real-time PCR with Ct values between 21 and 36 (data not shown) required about 32–54 min to obtain the final results.

**Fig. 3:**
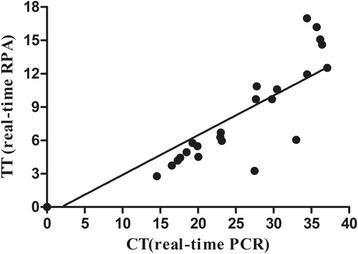
Comparisons between results of real-time RPA and real-time PCR on clinical samples. DNA extracts from 34 clinical samples were screened. Linear regression analysis of real-time RPA threshold time (TT) values (y axis) and real-time PCR cycle threshold (CT) values (x axis) were determined using Prism software. R^2^ value = 0.846

Results of the evaluation of exo RPA with clinical samples are shown in Table 2 and Fig. 3. The diagnostic performance of the real-time RPA assay to detect CPV-2 in the 91 clinical swab samples was compared to that of the real-time PCR. These two assays showed the same results (76 positive and 15 negative cases), and further analysis demonstrated that the real-time RPA had a diagnostic agreement of 100% with the real-time PCR (Table 3). No discrepancy was found in samples (14/76) containing low levels of CPV-2 DNA (Ct > 35, real-time PCR), indicating that the established real-time RPA reliably detected low amounts of CPV-2 in clinical samples. Positive samples had real-time RPA Ct values ranging from 17.52 to 36.29, indicating that the method was able to detect CPV-2 DNA across the entire range of the assay. Twenty-three positive samples were selected randomly, and the threshold time (TT) and cycle threshold (Ct) values of real-time RPA and real-time PCR, respectively, were well correlated with an R^2^ value of 0.846 (Fig. 3). The relative sensitivity of the real-time RPA assay was further evaluated by comparing with the SNAP test. A total of 30 fecal swab samples were collected for this experiment. Twenty-four of the above tested clinical samples displayed CPV-2 positive, six of them were CPV-2 negative by real-time PCR assay. The result showed that the SNAP test was able to detect CPV antigen in 16/30 (53.3%) of analyzed samples. A higher detection rate (24/30, 80%) was obtained using real-time RPA for CPV-2 DNA. The relative sensitivity of the SNAP test was 66.7% (16/24), when compared to real-time RPA. The best correlation was observed again between conventional real-time PCR and real-time RPA analysis (shown in the Additional file 1: Table S1). The detection results of the swab samples showed that the real-time RPA method we developed was effective in detecting CPV-2.

**Table 3:** Detection of CPV-2 in clinical samples by real-time RPA and real-time PCR

		Real-time PCR		
		Positive	Negative	Total
Real-time RPA	Positive	76	0	76
	Negative	0	15	15
	Total	76	15	91

## Discussion

In this study, a real-time RPA method was developed based on an exo probe for the rapid and sensitive detection of CPV-2. With real-time RPA, only CPV-2a and CPV-2b among multiple viruses infectious to canines tested could be amplified, demonstrating the high specificity of this assay (Fig. 1). Using the CPV-2 plasmid DNA as a template, it was detected between 4 and 12 min for 10^5^–10^1^ copies of DNA (Fig. 2a). The limit of detection of the real-time assay was 10^1^ copies/reaction, which was 10 times higher than that of the conventional RPA [18]. Further validation of this method was performed using clinical samples. The detection rate of the real-time RPA assay was comparable of that of the real-time PCR, although the former assay was much faster than the latter. The real-time RPA assay detected the positive samples within 4–12 min. While the CPV-2 detection results by real-time RPA could be obtained in about 30 min including the time for nucleic acid extraction, the reaction time for positive samples reached up to 1 h with real-time PCR. Additionally, the cost per reaction performed in the real-time RPA, real-time PCR assays and commercial SNAP tests including the cost of the reagents and the samples preparation was about 4.3, 6.0 and 5.0 US dollars, respectively. The cost of real-time RPA decreased 28.33%, when the method was compared to real-time PCR, and 14% respectively, when compared to SNAP tests. RPA has been widely explored for the molecular detection of diverse pathogens, including H7N9 virus and Dengue virus infection, and real field testing also has been achieved [23, 24]. Moreover, the portable POC tube scanner (Genie III, OptiGene Limited, West Sussex, United Kingdom) used in the study, weighing only 1.75 kg with the dimensions of 25 cm × 16.5 cm × 8.5 cm, is much more simple and less expensive than a real-time PCR machine and can be run on battery power for use in the field. For CPV-2, it is often sufficient to simply boil the fecal samples before running diagnostic PCR. In our subsequent experiments we obtained similar results using boiling only to extract CPV-2 from clinical samples. In recent years, a number of isothermal DNA amplification methods have been developed as a simple, rapid technique alternative to PCR-based amplification. In the LAMP assay for the rapid and sensitive detection of CPV-2, a set of four primers was needed, and the optimum time and temperature were 60 min and 65 °C, respectively [12]. The developed iiPCR could detect as low as 13 copies of CPV-2 DNA in about 60 min [9]. For the real-time RPA assay described in this work, 10^1^ copies of CPV-2 DNA could be detected in 4 min, which was much more rapid than the above assays, including the common RPA assay. Compared to other isothermal amplification techniques, RPA does not require initial heating for DNA denaturation, and the results can be obtained in less than 12 min. The major advantages of RPA compared with other isothermal DNA amplification methods are that it (1) shows a certain degree of tolerance to common PCR inhibitors, (2) can tolerate a wide range of biological samples [19] and (3) utilizes reagents stored in lyophilized pellets, which are very stable and can react satisfactory at 25 °C for up to 12 weeks and at 45 °C for up to 3 weeks [25]. The SNAP test based on ELISA protocols for rapid detection of viral antigen is commonly used for CPV-2 clinical diagnosis, but it is less sensitive than RCR-based methods, especially RAP assay we developed in this study. The lower sensitivity of the SNAP test is generally associated with the host immune response, such as the very small amounts of virus shed in the feces during the late stage of infection and/or the presence of high CPV antibody titres in the gut lumen and so on. In our RPA assay, the detecting target was the nucleic acid of CPV-2. The virus-specific gene was amplified first, and its amplification products were tested. In this process, the detection signal is magnified hundreds of thousands of times. More importantly, it is not affected by the host immune response. One limitation of our assay is that it fails to distinguish CPV-2 vaccine from wild type stains. If vaccine strain is shed post vaccination it will be detected by this assay. All in all, these distinguishing features of the RPA assay and hands-on experience of real field testing using RPA from other research groups make us believe that RPA could work well in a clinical diagnostic setting. It may also be the most applicable approach for the field and POC diagnosis of infectious diseases.

## Conclusion

In conclusion, the real-time RPA method based on an exo probe was successfully developed for the detection of CPV-2. With high sensitivity and specificity, the assay could be completed within 12 min. More importantly, the portability of the real-time RPA assay renders it applicable at quarantine stations, ports or sites of outbreaks. The effective and rapid real-time RPA assay developed in this study would be highly useful in the control of CPV-2, especially in resource-limited settings.

## Additional file

Additional file 1: Detection of CPV-2 in clinical samples by real-time RPA, real-time PCR and SNAP. (DOCX 14 kb)

## Abbreviations

BHK-21: Baby hamster kidney cells
CCoV: Canine coronavirus
CDV: Canine distemper virus
CPIV: Canine parainfluenza virus
CPV-2: Canine parvovirus 2
Ct: Cycle threshold
FP: Forward primer
iiPCR: Insulated isothermal PCR
LAMP: Loop-mediated isothermal amplification
LOD: Limit of detection
MDCK: Madin–darby canine kidney cells
n: Number
PCR: Polymerase chain reaction
POC: Point-of-care
PRV: Pseudorabies virus
RP: Reverse primer
RPA: Recombinase polymerase amplification
RT: Real-time reverse transcription
TT: Threshold time
