# Supplementary material for: Development of real-time recombinase polymerase amplification assay for rapid and sensitive detection of canine parvovirus 2
Source: BMC Vet Res. 2017 Nov 6;13:311. doi: 10.1186/s12917-017-1232-z (PMC5674863; doi:10.1186/s12917-017-1232-z)
Supplement: Additional file 1: — Detection of CPV-2 in clinical samples by real-time RPA, real-time PCR and SNAP. (DOCX 14 kb) [file 12917_2017_1232_MOESM1_ESM.docx]

Table S1. Detection of CPV-2 in clinical samples by real-time RPA, real-time PCR and SNAP

| Sample number | Detection results | | |
| --- | --- | --- | --- |
|  | real-time RPA  (TT) | real-time PCR  (Ct) | SNAP  (+/-) |
| 1 | 2.77 | 14.54 | + |
| 2 | 3.73 | 16.53 | + |
| 3 | ­- | >40 | - |
| 4 | 8.57 | 32.74 | - |
| 5 | 5.47 | 19.92 | + |
| 6 | 3.25 | 27.47 | + |
| 7 | ­- | >40 | - |
| 8 | 6.28 | 22.94 | + |
| 9 | 12.52 | 37.09 | - |
| 10 | 4.17 | 17.29 | + |
| 11 | ­11.93 | 34.44 | - |
| 12 | 5.75 | 19.28 | + |
| 13 | ­- | >40 | - |
| 14 | ­13.17 | 35.69 | - |
| 15 | ­9.70 | 27.65 | - |
| 16 | 5.95 | 23.14 | + |
| 17 | 10.85 | 27.76 | + |
| 18 | 9.03 | 32.99 | - |
| 19 | 6.70 | 23.00 | + |
| 20 | ­- | >40 | - |
| 21 | ­- | >40 | - |
| 22 | 10.58 | 30.46 | + |
| 23 | 9.70 | 29.79 | + |
| 24 | ­14.60 | 36.40 | - |
| 25 | 4.50 | 20.03 | + |
| 26 | 4.43 | 17.63 | + |
| 27 | ­- | >40 | - |
| 28 | 15.07 | 36.16 | - |
| 29 | 8.90 | 28.05 | + |
| 30 | 4.92 | 18.47 | + |

Note: ‘+’means positive; ‘-’means negative.

**Supplementary Methods**

**Detection of CPV-2 in clinical samples by real-time RPA, real-time PCR and SNAP parvo antigen test**

A total of 30 fecal swab samples were collected from the dogs sent to our laboratory from 2012 to 2016 and snap-frozen for storage at -80 ℃. Twenty-four of the above clinical samples had been tested to be CPV-2 positive, and six of them had been tested to be CPV-2 negative by real-time PCR. The SNAP test was carried out with the commercial kit (SNAP^®^ Canine Parvovirus Antigen Test, IDEXX Laboratories, Inc., Westbrook, Manine, USA), following the manufacturer’s instructions. The DNA extracts from a part of the fecal swab samples were tested by the real-time RPA and PCR method.

Sensitivity = true positives ÷ ( true positive ﹢false negatives )
